# Supplementary material for: Geographic Variations in Dietary Patterns and Their Associations with Overweight/Obesity and Hypertension in China: Findings from China Nutrition and Health Surveillance (2015–2017)
Source: Nutrients. 2022 Sep 23;14(19):3949. doi: 10.3390/nu14193949 (PMC9572670; doi:10.3390/nu14193949)
Supplement: Supplementary file 1 [file nutrients-14-03949-s001.zip › nutrients-1905937-supplementary.pdf]

File S1. The selection process of study participants.

In this study, we excluded 24,391 participants aged less than 45 years old, 3104 participants with no available data of blood pressure, body weight, and height, 4750 participants with abnormal intake of energy, cooking oil, and sodium, and 7730 participants with self-reported diagnosed cardiovascular disease (such as myocardial infarction). We further excluded 6780 participants who changed their diet due to obesity, or self-reported diagnosed hypertension, or other metabolic disease (e.g., self-reported hyperglycemia or hyperlipidemia) in past 12 months.

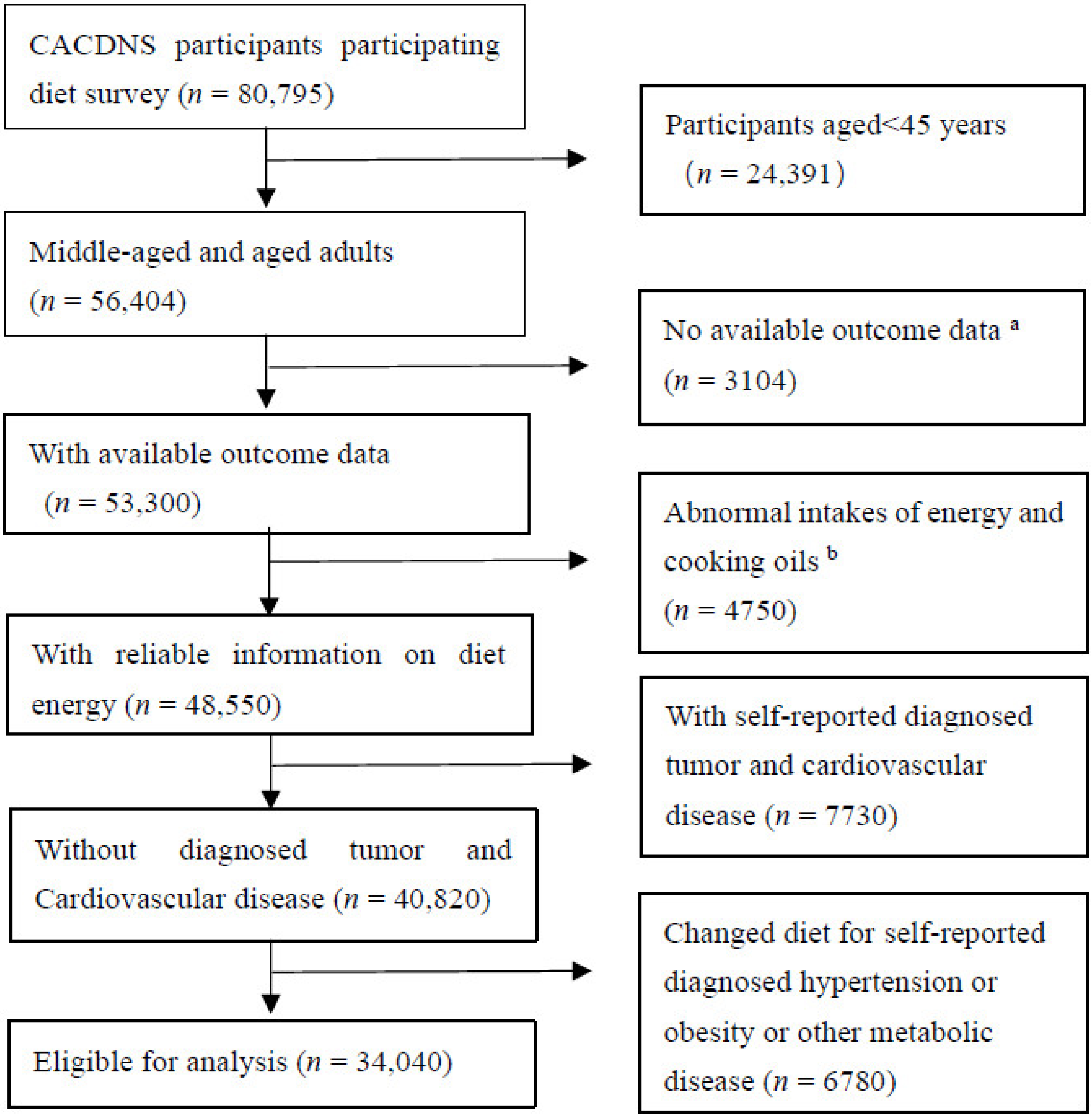

Figure S1. Participants selection flow chart. [a.] Outcome data included body weight, height, and blood pressure; [b.] Abnormal energy intake was <500 kcal or >4000 kcal; abnormal cooking oils was >150 g per day (nearly 99th percentile in survey population). CACDNS: China Adult Chronic Disease and Nutrition Surveillance.

File S2. Diet survey in field.

For each dietary recall day, trained investigators went to the participants' home and helped to recall and record food item and intake (eaten in home and outside of home) during the last 24-h. Investigators also weighed the household cooking oil and condiments, and recorded the number of diners (including guests) at each meal in home as well as their sex, age, and physical activity level at the beginning and end of each 24-hour survey. These information was used to allocate the 3-day consumption proportion of cooking oil and condiments for each participant in the household, and calculate their actual number of meals and consumption during the 3 days. At the end of each 24-h survey, trained investigators coded the collected food items according to the food composition table (FCTS) via PAD-aid investigation technology.

File S3. Definitions of food groups and nutrients, and methods for calculating intake.

In present study, we classified 2270 food items in the constantly updating food composition database into 35 food groups, including refined grains, coarse grains, rice, wheat, corn, mixed beans, other grains, tuber, vegetables, dark green vegetables, red vegetables, starchy vegetables, legumes, fungi and algae, salted pickle, chili, spices and herbs, other vegetables, fruits, nut and seeds, dairy, soybeans, eggs, red meat, poultry, fish, offal, processed meat, high-sugar foods, salty snacks, beverages, high MUFA oils, MUFA-PUFA balanced oil, high PUFA oils, and high SFA fats, of which mixed beans, spices and herbs, salty snacks, and beverages were consumed by less than 5% of study population, and was excluded in later dietary pattern analysis. FCTs 2009 and 2018 were the reference for classification of food groups [30,31]. In addition, several new classifications for food groups and nutrients were introduced based on the research literature on the relationship between diet and cardiometabolic diseases, as shown in Table S1.

File S4. The correlations of food group intakes between 3 consecutive days of 24-h diet recalls and FFQ.

To evaluate the correlation of short term diet assessed by 3 consecutive days of 24-h diet recalls with long term diet assessed by FFQ in our participants, we conducted a spearman correlation test with food groups assessed by both of the two methods. Since the FFQ did not assess the intakes of cooking oils and various types of vegetables, we assessed the correlation of other major food groups. The correlation coefficients between food groups ranged from the lowest 0.1 for processed meat to 0.65 for wheat. Overall, staple grains and fish had relatively higher correlation coefficients. However, the median intake assessed by the two methods was quite different, especially for rice, fruits, and eggs. Our result was comparable with previous assessment for 2002 China National Nutrition and Health Survey [89]. See Table S2.

Table S1. Definitions of selected food groups and nutrients and methods for calculating intakes.

Food groups or nutrients	Definitions	Methods for calculating intake
Coarse grain	A portion of the outer layer of the grain seed is retained, but not as nutritionally complete as the whole grain, such as crushed corn dregs and millet.	$\text{Actual consumption of each food item} \times \frac{\text{Carbonhydrate content of each food item (per 100 gram)}}{\text{Carbonhydrate content of raw wheat (per 100 gram)}}$ Units were calculated in grams.
Refined grain	Grains or grain products with the bran and germ removed, low in fiber. Included flour, starch, rice, noodles, instant breakfast cereals/nachos, bread, pasta/cookies and baked goods/desserts.	Same as above except with flour as reference.
High-quality carbohydrates	Food rich in dietary fibers, including grains with fiber content exceeding 1 g per 100 kcal, fresh fruits, and vegetables (including potatoes and starchy vegetables).	Actual consumption of each food item × carbohydrate content for that item (per 100 grams) Units were calculated in grams.
Other grain	Grains except rice, wheat, and corn, and their products, such as millet, buckwheat, and barley.	Same as above.
Tubers	Including potatoes and sweet potatoes, which can be eaten as staple in Chinese cuisine.	$\text{Actual consumption of each food item} \times \frac{\text{Water content of each food item (per 100 gram)}}{\text{Water content of sweet potatoes (per 100 gram)}}$
Starchy vegetables	Vegetables having a high carbohydrate content except all potatoes, such as lotus root, water chestnut, and Chinese yam	Actual consumption. Units in grams.
High-sugar foods	Referred to cakes, pastries, cookies, ice cream and candies with added sugar content exceeding 15 g per 100 g.	$\text{Actual consumption of each food item} \times \frac{\text{Energy of each food item (per 100 grams)}}{\text{Energy of green bean cake (per 100 grams)}}$
Processed meat	Meat that has been transformed through salting, curing, fermentation, smoking, or other processes to enhance flavor or improve preservation, particularly those industrially processed [34].	Same as above except with Chinese traditional sausage as reference
High-MUFA oils	Oils with the proportion of monounsaturated fatty acids exceeding 50 percent of total fat content, such as rapeseed oil, tea-seed oil and olive oil.	Actual consumptions. Units in grams.
MU-PUFA balanced oils	Oils with the proportions of monounsaturated and polyunsaturated fatty acids both ranging between 30 and 50 percent of total fat content, such as sesame oil, salad oil and peanut oil.	Same as above.
High-PUFA oils	Oils with the proportions of polyunsaturated fatty acids exceeding 50 percent of total fat content, such as soybean oil, corn oil, sunflower oil and walnut oil.	Same as above.
High-SFA fats	Fats with the proportions of saturated fatty acids exceeding 40 percent of total fat content, such as lard, butter and coconut oil.	Same as above.

Abbreviation: MUFA: monounsaturated fatty acids; PUFA: polyunsaturated fatty acids; SFA: saturated fatty acids.

Table S2. Comparison of intakes of major food groups assessed by 3 consecutive days of 24-h diet recalls and FFQ and the correlation coefficients between the two methods in study population *.

Food groups	Diet recalls	FFQ	Spearman coefficients
Coarse grain	0.0 (0.0,0.0)	1.3(0.0,19.5)	0.42
Refine grain	163.8 (106.6,243.6)	272.0 (171.4,402.9)	0.20
Rice	61.0 (24.8,132.5)	135.3 (38.7,270.7)	0.59
Wheat	48.8 (8.8,117.2)	48.8 (8.7,143.6)	0.65
Corn	0.0 (0.0,0.0)	0.0 (0.0,6.5)	0.44
Other grain	0.0 (0.0,0.0)	0.0 (0,0.5)	0.50
Tuber	0.0 (0.0,53.1)	13.7 (1.6,47.0)	0.38
Vegetables	216.7 (135.0,316.7)	250.0 (122.8,416.9)	0.32
Fruits	0.0 (0.0,32.9)	42.8 (10.0,107.1)	0.30
Soybeans	0.5 (0.0,12.3)	5.7 (1.5,14.4)	0.26
Red meat	45.8 (11.7,92.0)	36.2 (13.6,90.7)	0.37
Poultry	0.0 (0.0,0.0)	2.0 (0.0,8.0)	0.25
Fish	0.0 (0.0,29.5)	5.7 (0.1,20.5)	0.47
Offal	0.0 (0.0,0.0)	0.0 (0.0,0.3)	0.15
Processed meat	0.0 (0.0,0.0)	0.0 (0.0,0.1)	0.10
Dairy	0.0 (0.0,0.0)	0.0 (0.0,11.0)	0.34
Eggs	3.3 (0.0,32.3)	20.5 (7.0,50.0)	0.32
High-sugar foods	0.0 (0.0,0.0)	0.0 (0.0,5.4)	0.15

*. Intakes was described as median (interquartile range). Abbreviation: FFQ for food frequency questionnaire.

File S5. Age-sex-standardized prevalence of overweight/obesity and hypertension for four dietary patterns.

As Table S3 shown, people adopting NWB had highest prevalence of overweight/obesity, and hypertension. People adopting SRB had lowest prevalence of overweight/obesity, while people adopting PD had the lowest prevalence of hypertension.

Table S3. Age-sex-standardized prevalence of overweight/obesity, and hypertension for four dietary patterns among study population *.

	Overall	CRB	PD	NWB	SRB	*p* value
No. of participants	34,040	13,907	3806	8992	7335	
Overweight/obesity, %	47.7 (0.76) [a]	45.6 (1.04)	47.4 (1.31)	55.9 (1.30)	41.0 (1.30)	<0.001
Hypertension, %	44.6 (0.94)	45.0 (1.20)	38.7 (1.53)	49.4 (1.30)	40.6 (1.41)	<0.001

*. Rao Scott Chi-Square test were used to test the between-group differences. [a]. Prevalence rate (standard error). Abbreviation: CRB: common rice-based dietary pattern; PD: prudent diversified dietary pattern; NWB: northern wheat-based dietary pattern; SRB: southern rice-based dietary pattern; No.: number.

File S6. Mean intakes of selected food groups for four diet patterns.

Data presented in Figure 3 were from Table S4. Significant differences in food group intakes among various dietary patterns were detected.

Table S4. Mean intakes of selected food groups for four diet patterns among study population (g/1000 kcal) *.

Food groups	Overall	CRB	PD	NWB	SRB	*p* value
Coarse grain	6.7	1.3	12.3	15.7	3.1	<0.001
Refine grain	121.5	124.5	103.8	131.9	112.4	<0.001
Rice	60.1	76.4	51.3	17.6	85.5	<0.001
Wheat	52.4	41.0	47.2	97.0	21.8	<0.001
Corn	5.0	0.6	0.0	15.4	3.1	<0.001
Other grains	4.2	1.4	11.3	8.9	0.0	<0.001
Tubers	26.8	26.7	24.1	33.2	20.5	<0.001
Vegetables	167.4	172.3	163.4	141.3	192.3	<0.001
Dark green vegetables	53.7	61.4	56.9	26.7	70.7	<0.001
Red vegetables	15.4	15.1	16.2	15.8	15.1	<0.001
Starchy vegetables	7.0	0.1	13.5	5.2	19.1	<0.001
Other vegetables	78.9	82.3	63.2	83.9	74.3	<0.001
Fruits	18.7	16.6	30.0	19.4	16.2	<0.001
Soybeans	6.4	7.0	6.5	5.3	6.6	<0.001
Red meat	39.5	44.0	39.0	27.7	45.8	<0.001
Poultry	8.4	9.9	10.9	1.6	12.4	<0.001
Fish	15.9	19.7	18.8	5.1	20.4	<0.001
Offal	1.8	0.0	4.8	0.4	5.2	<0.001
Processed meat	1.8	0.0	4.3	0.2	5.8	<0.001
Dairy	10.1	0.2	34.3	21.1	2.9	<0.001
Eggs	12.6	11.8	14.3	14.8	10.5	<0.001
High-sugar foods	3.0	2.8	6.6	3.0	1.8	<0.001
Oils and fats						
High MUFA	3.1	3.9	7.0	2.5	0.3	<0.001
MUFA-PUFA balance	2.8	2.8	0.4	3.1	3.4	<0.001
High PUFA	3.1	3.7	4.3	4.1	0.1	<0.001
High SFA	1.5	0.5	0.0	0.0	5.8	<0.001

* Kruskal-Wallis test was used to compare the between-group differences.

File S7. Recommended intakes of food groups and nutrients in Figure 4.

Balanced Dietary Pattern recommended in Chinese Dietary Guideline (2022) were the references of the recommended intakes for dietary components [38]. Density method, or amount of dietary components per 1000 kcal, was adopted to set the recommend values [39]. The recommended intakes for food groups adopted least-restrictive recommendation among the 1600 kcal to 2400 kcal patterns. The criterion of excessive intakes for SFA and sodium adopted the upper recommendation, while for red meat and oils it was set at intake ≥200% the upper recommendation [40]. See Table S4 for detail.

Table S5. Criterion of recommended intakes and excessive intakes for dietary components *.

Dietary components	Recommended intakes	Criterion of excessive intakes
Grains	125	-
Coarse grains	30	-
Tubers	30	-
Vegetables	200	-
Dark green vegetables	100	-
Red meat	15	≥35
Poultry	10	-
Fish	15	-
Eggs	20	-
Dairy	125	-
Soybeans	5	-
Nuts	5	-
Oils	12-15	≥25
Sodium	<2	≥2
SFA, E%	<10	≥10

* Data expressed as grams per 1000 kcal unless otherwise indicated. Abbreviations: SFA: saturated fatty acids.

File S8. The robustness of the characteristics of derived dietary patterns.

To test the robustness of the characteristics of four dietary patterns identified by finite mixture model, we conducted a split-sample validation. First, we split the data into two random samples. Then, we re-derived dietary patterns separately in these two samples, and compared their characteristics of food group intake. The food group intake of re-derived dietary patterns in each sample were shown in Figure S2. The two samples showed similar food intake patterns with the whole sample.

(a)

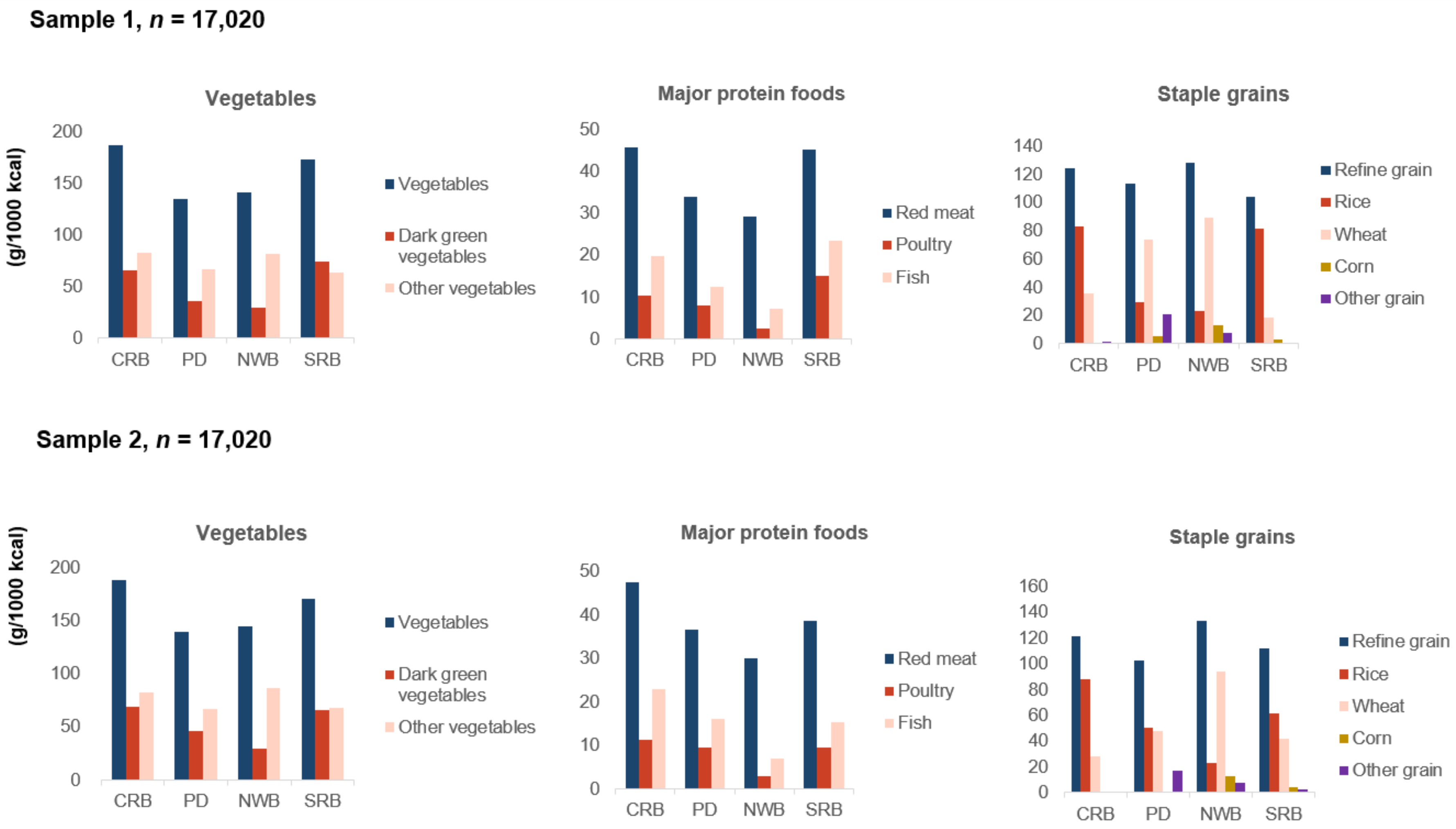
Sample 1, n = 17,020
Vegetables
(g/1000 kcal)
Vegetables
Dark green vegetables
Other vegetables
Major protein foods
Red meat
Poultry
Fish
Staple grains
Refine grain
Rice
Wheat
Corn
Other grain
CRB
PD
NWB
SRB
Sample 2, n = 17,020
Vegetables
(g/1000 kcal)
Vegetables
Dark green vegetables
Other vegetables
Major protein foods
Red meat
Poultry
Fish
Staple grains
Refine grain
Rice
Wheat
Corn
Other grain
CRB
PD
NWB
SRB

(b)

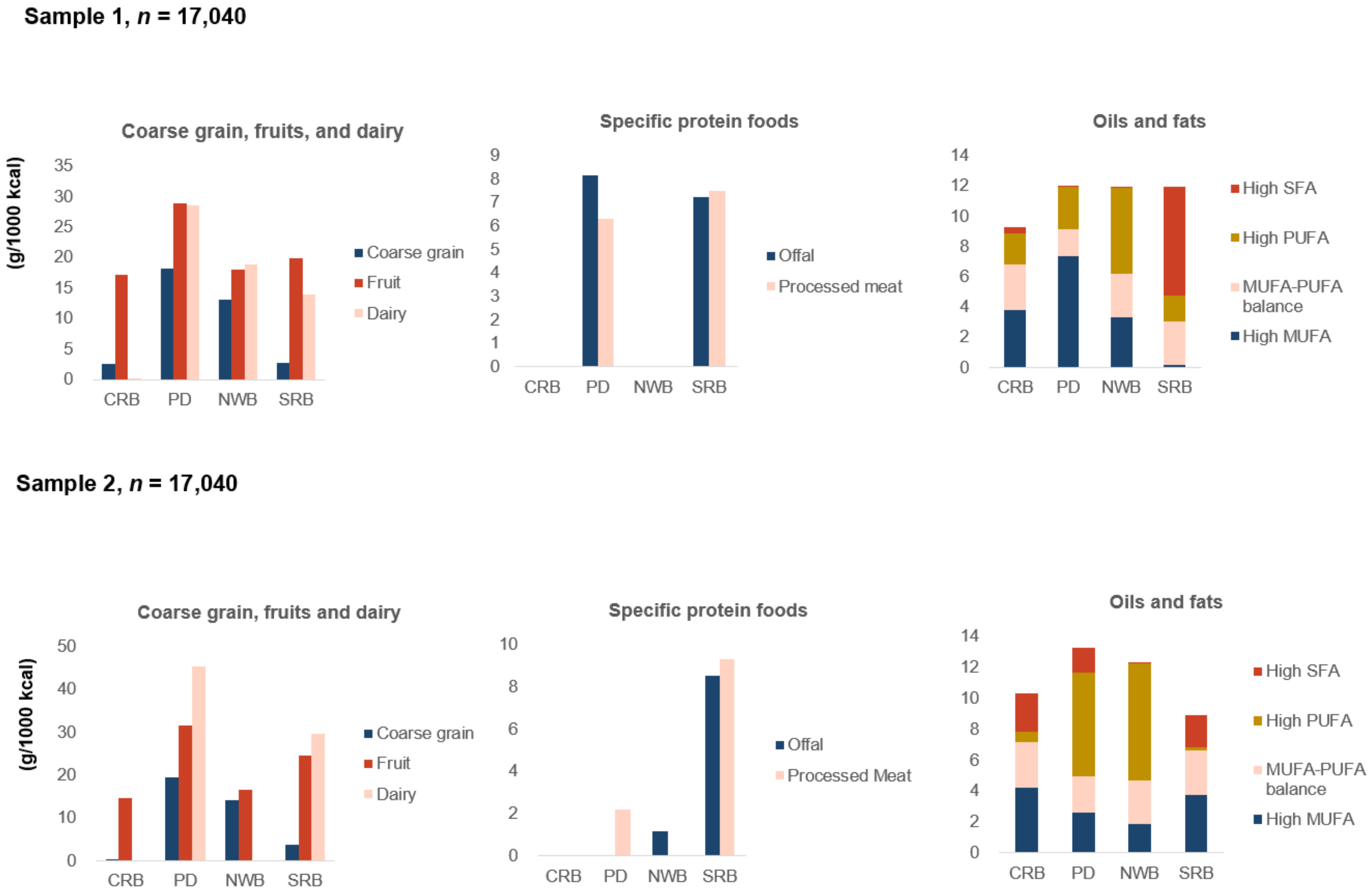

Figure S2. (**a**) Mean intakes of selected food groups for re-derived dietary patterns (**a**). Intake was calculated as grams per 1000 kcal energy. (**b**) Mean intakes of selected food groups for re-derived dietary patterns (**b**). Intake was calculated as grams per 1000 kcal energy. Abbreviation: CRB: common rice-based dietary pattern; PD: prudent diversified dietary pattern; NWB: northern wheat-based dietary pattern; SRB: southern rice-based dietary pattern; SFA: saturated fatty acids; PUFA: polyunsaturated fatty acids.

Table S6. Age-sex-standardized proportions of four diet patterns among study population by 31 provinces in urban and rural areas.

Geographic division	Province	No. of participants	Overall				Urban				Rural				*p* value *
			CRB	PD	NWB	SRB	CRB	PD	NWB	SRB	CRB	PD	NWB	SRB	
North	Beijing	619	28.0 (2.95) [a]	17.2 (1.13)	51.9 (2.49)	2.9 (0.87)	25.8 (3.60)	19.6 (0.74)	52.4 (3.42)	2.2 (0.69)	37.2 (3.89)	7.5 (3.01)	49.4 (1.78)	5.9 (2.56)	0.001
	Tianjin	492	26.4 (4.18)	4.8 (1.33)	63.5 (5.41)	5.3 (1.73)	20.2 (2.99)	5.4 (1.34)	68.5 (4.11)	5.9 (2.01)	53.3 (7.74)	2.1 (2.04)	42.1 (7.62)	2.6 (0.47)	<0.001
	Hebei	1529	22.0 (3.51)	10.7 (5.22)	63.8 (7.94)	3.5 (0.43)	20.7 (5.37)	10.9 (7.45)	66.5 (11.67)	1.8 (0.62)	22.8 (3.68)	10.6 (6.03)	61.9 (8.53)	4.7 (0.76)	0.843
	Shanxi	987	10.9 (2.35)	18.3 (1.53)	70.4 (2.46)	0.5 (0.26)	12.8 (6.41)	16.3 (2.67)	69.6 (5.85)	1.3 (0.22)	9.8 (1.29)	19.3 (0.68)	70.8 (1.29)	0.1 (0.1)	0.463
	Inner Mongolia	743	40.7 (5.39)	12.8 (3.65)	34.4 (10.11)	12.2 (6.61)	44.3 (3.63)	14.1 (6.32)	33.9 (4.47)	7.7 (5.18)	37.3 (9.29)	11.6 (3.14)	34.8 (15.8)	16.3 (9.41)	0.612
Northeast	Liaoning	1112	50.2 (6.28)	17.6 (3.54)	25.0 (4.96)	7.2 (1.96)	48.9 (10.17)	20.0 (3.47)	25.4 (7.01)	5.7 (1.95)	52.3 (5.12)	13.8 (6.01)	24.4 (3.49)	9.6 (3.39)	0.699
	Jilin	826	74.2 (2.57)	4.6 (1.22)	13.6 (1.61)	7.6 (1.31)	73.4 (5.32)	8.1 (2.66)	13.5 (3.19)	5 (2.45)	74.9 (1.69)	1.5 (0.88)	13.6 (1.00)	10.0 (1.47)	0.031
	Heilongjiang	1022	47.0 (8.62)	13.2 (3.04)	33.9 (8.78)	5.8 (1.33)	32.4 (5.32)	20.7 (6.11)	43.2 (10.57)	3.7 (1.02)	64.7 (6.44)	4.3 (1.12)	22.7 (3.84)	8.3 (2.72)	<0.001
East	Shanghai	690	41.4 (5.11)	37.5 (4.91)	10.8 (2.40)	10.3 (1.68)	41 (5.29)	38.9 (4.24)	11.9 (2.19)	8.1 (0.85)	44.2 (3.82)	26.6 (5.06)	2.3 (1.58)	26.9 (2.82)	<0.001
	Jiangsu	1465	42.7 (7.59)	12.3 (2.19)	20.6 (8.29)	24.4 (4.16)	44.9 (8.04)	17.1 (3.42)	13.7 (5.51)	24.2 (2.89)	40.5 (9.09)	7.4 (2.91)	27.6 (12.5)	24.6 (4.82)	0.038
	Zhejiang	1308	44 (3.41)	19.4 (1.86)	6.0 (0.86)	30.6 (1.86)	35.9 (3.84)	26.8 (3.51)	10.2 (2.16)	27.2 (2.43)	51.6 (2.01)	12.5 (1.51)	2.2 (0.76)	33.8 (1.2)	<0.001
	Anhui	1610	61.1 (3.56)	9.6 (1.28)	8.7 (3.19)	20.6 (4.81)	56.2 (4.54)	11.9 (2.42)	11.2 (4.48)	20.8 (5.85)	64.2 (4.01)	8.2 (1.12)	7.2 (2.67)	20.4 (4.98)	0.169
	Fujian	1217	49.6 (7.43)	12.1 (1.81)	2.2 (0.65)	36.0 (7.47)	42.2 (3.88)	17.7 (3.53)	4.0 (0.91)	36.1 (2.56)	55.7 (12.98)	7.6 (2.07)	0.8 (0.74)	35.9 (12.14)	0.113

	Jiangxi	1480	46.2 (5.99)	11.3 (2.91)	2.7 (1.38)	39.7 (5.81)	42.6 (9.46)	17.9 (4.54)	5.8 (3.18)	33.8 (4.57)	48.5 (5.66)	7.1 (2.57)	0.7 (0.51)	43.6 (7.88)	0.003
	Shandong	1575	18.9 (2.62)	6.3 (1.98)	71.9 (3.79)	2.9 (0.72)	18.4 (3.29)	8.1 (3.94)	70.4 (6.79)	3.1 (0.61)	19.3 (3.55)	5.0 (1.47)	72.9 (4.71)	2.8 (1.18)	0.781
Central	Henan	1477	16.8 (7.56)	2.8 (1.08)	77.6 (7.83)	2.8 (1.11)	17.3 (9.59)	5.1 (2.51)	73.3 (10.43)	4.4 (1.47)	16.7 (6.10)	1.8 (0.43)	79.4 (6.14)	2.1 (1.05)	0.073
	Hubei	1192	35.6 (4.78)	11.5 (1.35)	19.5 (2.92)	33.4 (4.16)	28 (3.36)	10 (1.57)	24.3 (2.19)	37.6 (2.98)	40.9 (5.58)	12.5 (1.74)	16.1 (3.37)	30.5 (5.42)	<0.001
	Hunan	1806	44.7 (4.99)	12.8 (1.82)	1.6 (0.71)	40.9 (4.04)	41.5 (3.10)	16.3 (2.83)	3.2 (1.48)	39 (4.65)	46.8 (6.92)	10.7 (1.61)	0.5 (0.37)	42.1 (5.94)	0.048
South	Guangdong	1823	52.4 (4.74)	8.7 (2.21)	1.5 (0.60)	37.4 (3.43)	46.8 (6.46)	12.0 (2.96)	2.3 (1.16)	38.9 (4.94)	59.8 (2.45)	4.3 (0.79)	0.4 (0.30)	35.4 (2.79)	<0.001
	Guangxi	1244	52.1 (8.64)	6.6 (1.59)	1.9 (0.65)	39.4 (7.05)	33.6 (7.41)	19.4 (1.35)	5.9 (0.82)	41.1 (7.48)	59.3 (9.34)	1.7 (0.53)	0.3 (0.24)	38.8 (8.75)	<0.001
	Hainan	788	57.8 (1.56)	9.1 (3.41)	2.6 (0.60)	30.4 (4.75)	56.9 (2.45)	13.8 (6.41)	4.1 (0.75)	25.3 (9.61)	58.6 (2.68)	5.5 (0.98)	1.4 (0.39)	34.5 (1.87)	<0.001
Southwest	Chongqing	1053	51.1 (2.58)	13.4 (3.69)	3.6 (1.15)	31.9 (2.93)	48.2 (4.82)	16.7 (6.25)	5.2 (1.84)	29.9 (5.45)	53.7 (2.92)	10.3 (4.42)	2.2 (1.17)	33.7 (3.46)	0.490
	Sichuan	1712	50.6 (6.61)	14.3 (1.39)	5.2 (2.81)	29.9 (4.88)	43.4 (9.27)	17 (2.77)	8.6 (5.67)	31.0 (5.01)	55.0 (7.43)	12.6 (1.72)	3.2 (1.01)	29.1 (6.46)	0.139
	Guizhou	835	53.5 (6.42)	3.3 (2.11)	3.1 (1.35)	40.1 (4.66)	67.2 (5.57)	3.0 (2.49)	1.6 (1.75)	28.2 (7.15)	48.9 (7.53)	3.5 (2.52)	3.6 (1.58)	44.0 (5.49)	0.205
	Yunnan	1253	31.6 (4.65)	12.4 (1.48)	1.4 (0.53)	54.6 (3.73)	39.3 (8.93)	16.9 (2.96)	2.2 (1.55)	41.7 (7.15)	28.5 (3.84)	10.6 (2.3)	1.0 (0.59)	59.9 (1.8)	0.057
	Tibet	305	10.6 (5.08)	20.3 (3.68)	69.1 (5.32)	0	16.5 (0)	16.8 (0)	66.7 (0)	0	9.1 (3.28)	21.2 (5.24)	69.7 (5.99)	0	0.811
Northwest	Shaanxi	1145	16.7 (4.23)	4.1 (1.25)	76.8 (5.67)	2.4 (1.39)	18.6 (3.47)	4.6 (1.17)	76.0 (4.28)	0.9 (0.63)	15.5 (5.54)	3.8 (1.59)	77.3 (7.52)	3.4 (1.81)	0.222
	Gansu	828	40.8 (0.86)	9.9 (1.59)	48.4 (1.02)	0.9 (0.18)	56.2 (7.27)	7.0 (2.1)	36.2 (5.5)	0.7 (0.36)	34.1 (1.17)	11.1 (1.99)	53.7 (1.27)	1.0 (0.17)	0.001

Qinghai	604	18.2 (2.54)	12.6 (2.45)	68.8 (2.51)	0.4 (0.22)	17.7 (0.14)	19.1 (0.27)	62.5 (0.14)	0.7 (0.26)	18.6 (4.56)	7.1 (0.08)	74.3 (4.45)	0.1 (0.04)	0.340
Ningxia	634	46.0 (8.37)	11.3 (4.20)	38.1 (4.47)	4.6 (2.03)	28.3 (0)	18.6 (0)	44.6 (0)	8.5 (0)	59.2 (5.26)	5.8 (2.14)	33.3 (4.39)	1.7 (0.91)	<0.001
Xinjiang	666	31.0 (3.61)	8.4 (3.96)	58.9 (1.80)	1.7 (0.53)	22.7 (1.91)	15.5 (3.92)	60.5 (1.72)	1.2 (1.09)	38.0 (3.20)	2.5 (1.22)	57.5 (2.05)	2.1 (0.89)	<0.001

*. Rao Scott Chi-Square test were used to test the rural-urban differences in the proportion of four dietary patterns by 31 provinces. For provinces where the number of participants adopting specific dietary pattern was too small to calculate statistical parameters, we combined the participants adopting this pattern with participants adopting similar dietary pattern, for example, participants adopting SRB were combined with participants adopting CRB. [a.] Data described as proportion (standard error).

File S9. Dietary patterns derived with conventional methods, and their geographical distributions, as well as their associations with investigated outcomes.

Four patterns were named wheat-based dietary pattern (WB), dietary pattern featured by coarse grains (CG), rice-based dietary pattern (RB), and diversified dietary patterns (D), according to factor loadings of food groups, as distinctions to the dietary patterns in the main text. See Figure S3.

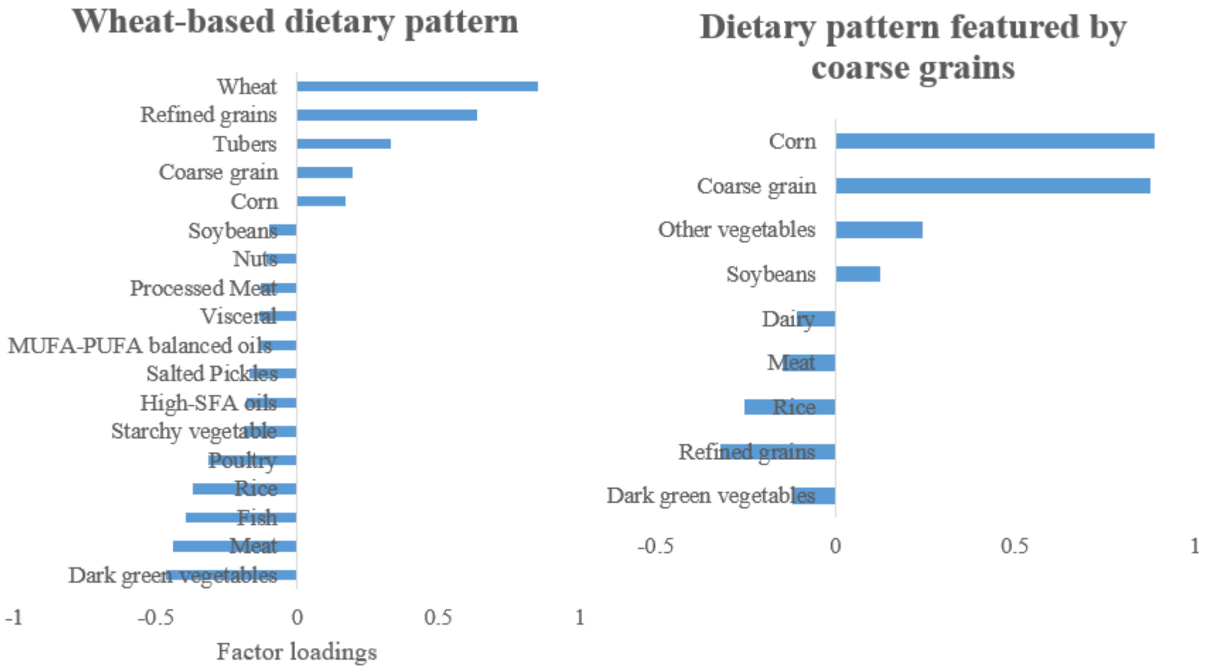

Figure S3. Characteristics of newly-derived dietary patterns. Food groups with absolute values of factor loadings less than 0.1 were not displayed. Figure S4 showed similar north-south distribution disparities of newly-derived dietary patterns with the dietary patterns in the main text. It's noteworthy that the CG geographically partly overlapped with the WB, indicating the plausibility of the existence of NWB (high consumption of both wheat and coarse grains) in the main text.

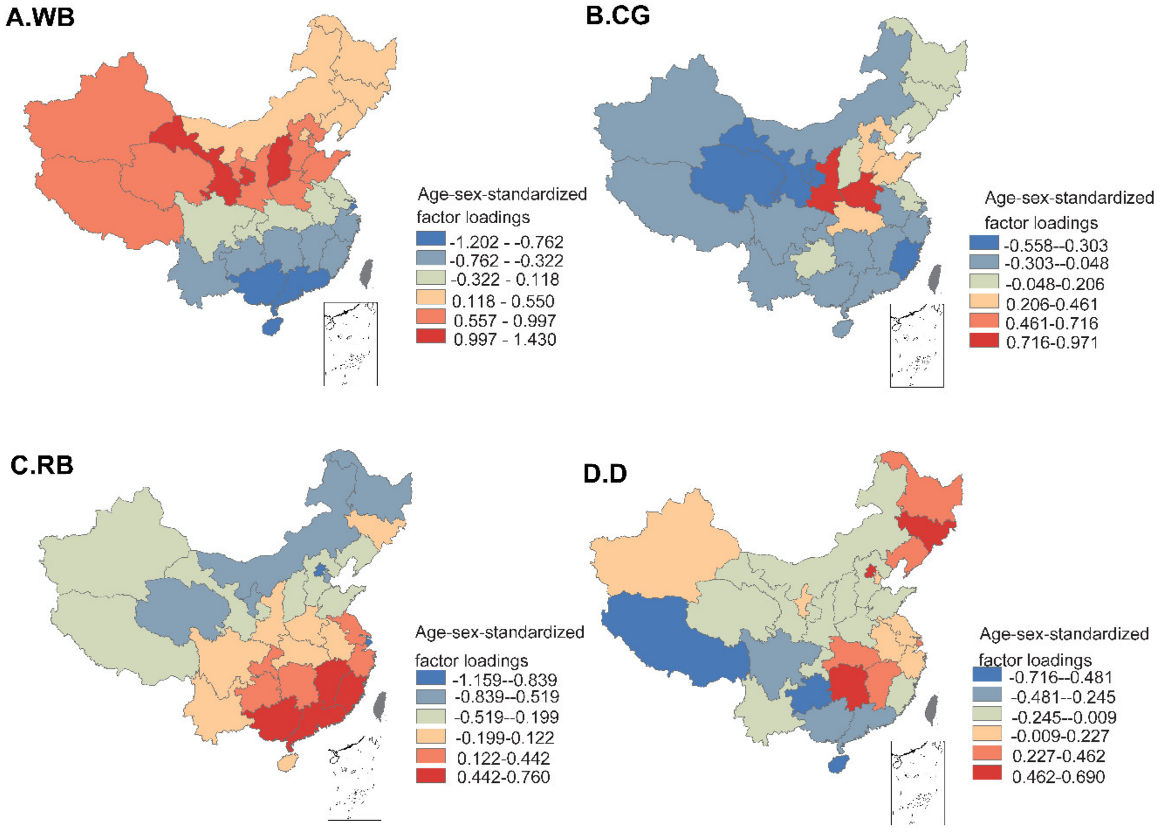

Figure S4. Geographic variations of newly-derived dietary patterns. WB: wheat-based dietary pattern; CG: dietary pattern featured by coarse grains; RB: rice-based dietary pattern; D: diversified dietary pattern.

As Table S7 shown, WB and CG were both positively associated with overweight/obesity (Q4 vs. Q1, ORs = 1.716 and 1.163, 95%CIs: 1.602–1.839 and 1.09–1.242, respectively, P-trend <0.001 for both) and hypertension (Q4 vs. Q1, ORs = 1.309 and 1.123, 95%CIs:1.219–1.405 and 1.050–1.200, respectively, P-trend <0.001 for WB and P-trend = 0.004 for CG), similar to NWB in the main text. RB was negatively associated with overweight/obesity but positively associated with hypertension (P-trend < 0.001 and P-trend = 0.002, respectively), which was different from SRB in the main text. The D was not associated with overweight/obesity despite the declining trend of risk of overweight/obesity across quartiles of D, yet, D was negatively associated with hypertension (Q4 vs. Q1, OR = 0.874, 95%CI: 0.816–0.936), which is comparable to PD in the main text, although they were slightly different in characteristic dietary components and geographic distribution.

Table S7. Associations of newly-derived dietary patterns with overweight/obesity and hypertension *.

	**Overweight/obesity**		**Hypertension**	
	OR (95%CI)	P for trend	OR (95%CI)	P for trend
Wheat-based dietary pattern		<0.001		<0.001
Q1	1.000		1.000	
Q2	1.176 (1.104–1.253)		1.071 (1.003–1.143)	
Q3	1.545 (1.448–1.648)		1.216 (1.138–1.300)	
Q4	1.716 (1.602–1.839)		1.309 (1.219–1.405)	
Dietary pattern featured by coarse grains		<0.001		0.004
Q1	1.000			
Q2	1.105 (1.036–1.179)		1.052 (0.984–1.125)	
Q3	1.125 (1.05–1.204)		1.106 (1.031–1.187)	
Q4	1.163 (1.09–1.242)		1.123 (1.050–1.200)	
Rice-based dietary pattern		<0.001		0.002
Q1	1.000			
Q2	0.959 (0.9–1.021)		1.077 (1.009–1.15)	
Q3	0.890 (0.834–0.949)		1.125 (1.052–1.203)	
Q4	0.785 (0.734–0.84)		1.118 (1.043–1.199)	
Diversified dietary pattern		0.007		<0.001
Q1	1.000			
Q2	1.080 (1.014–1.151)		0.908 (0.851–0.969)	
Q3	1.049 (0.983–1.119)		0.881 (0.825–0.942)	
Q4	0.976 (0.913–1.043)		0.874 (0.816–0.936)	

*. All models adjusted for were adjusted for sex, age, ethnic group, education level, household income per capita, occupation, smoking behavior, drinking behavior, physical activity, total energy intake, and family history of cardiovascular disease and diabetes, and body mass index were in addition adjusted for hypertension.

File S10. Socioeconomic and lifestyle characteristics of participants and their associations with dietary patterns.

Among our participants, female accounted for 50.8%, only 2.8% of them had an education level of college and above, 56.2% of them were engaged in agriculture and 27.6% were unemployed, 15.1% of them had a household income per capita of 40,000 Yuan and above, and 64.2% of them lived in rural areas. Among the participants classified as CRB, 53.2% were female, 67.2% were rural residence, 51.7% were engaged in agriculture and 26.0% were unemployed, 2.7% had an education level of college and above, and 16.6% had an annual per capita income of more than 40,000 Yuan. Most participants were never smoker, but nearly a quarter of them were moderate drinker.

Table S8. Characteristics of participants by dietary pattern groups *.

**Characteristics**		**Overall**	**CRB**	**PD**	**NWB**	**SRB**
Age		58.8	59.2	58.7	58.4	59.0
Sex	Female	50.8	53.2	59.7	50.9	41.5
	Male	49.2	46.8	40.3	49.1	58.6
Education	Iliterate	19.6	21.7	16.5	18.2	18.8
	Primary school	40.3	42.5	34.7	34.4	46.4
	Middle school	26.9	24.8	28.5	31.4	24.5
	High school	10.4	9.0	14.6	12.1	8.6
	College and above	2.8	2.0	5.7	3.8	1.6
Occupation	Not employed	27.6	26.4	36.9	29.2	23.0
	Agriculture	52.6	54.4	39.2	53.3	55.2
	Manufacture	3.0	3.0	3.1	2.2	3.8
	Service	8.3	7.2	12.7	7.9	8.4
	Others	8.6	9.0	8.2	7.4	9.6
Annual household income per capita	< ¥ 4000	16.3	16.4	11.3	18.2	16.6
	¥ 4000–9999	23.6	23.2	20.1	24.1	25.6
	¥ 10,000–24,999	32.1	32.9	33.6	32.0	30.2
	¥ 25,000–39,999	12.8	12.2	17.3	12.4	12.1
	¥ 40,000 and above	15.1	15.4	17.6	13.3	15.5
Sleep time (h)	7–9	53.9	54.3	57.0	53.6	51.8
	<7	21.5	22.5	21.2	20.8	20.8
	>9	24.6	23.2	21.8	25.6	27.5
Residence	Rural	64.2	67.2	51.1	61.4	68.7
	Urban	35.8	32.8	48.9	38.6	31.3
Smoking behavior	Never	63.4	65.3	70.0	65.3	54.1
	Ever	7.4	6.7	6.6	7.6	8.6
	Current	29.3	28.0	23.3	27.1	37.3
Drinking behavior	Never	62.8	64.7	64.4	64.0	57.0
	Moderate	25.7	24.1	26.0	26.7	27.5
	Excessive	11.5	11.2	9.6	9.3	15.5
Physical activity	Q1	24.9	24.2	24.1	28.7	22.1
	Q2	24.9	24.5	29.4	26.1	21.8
	Q3	24.9	25.6	24.5	23.8	25.3
	Q4	24.9	25.6	22.0	21.3	30.8

*. Data were expressed as proportions unless otherwise indicated.

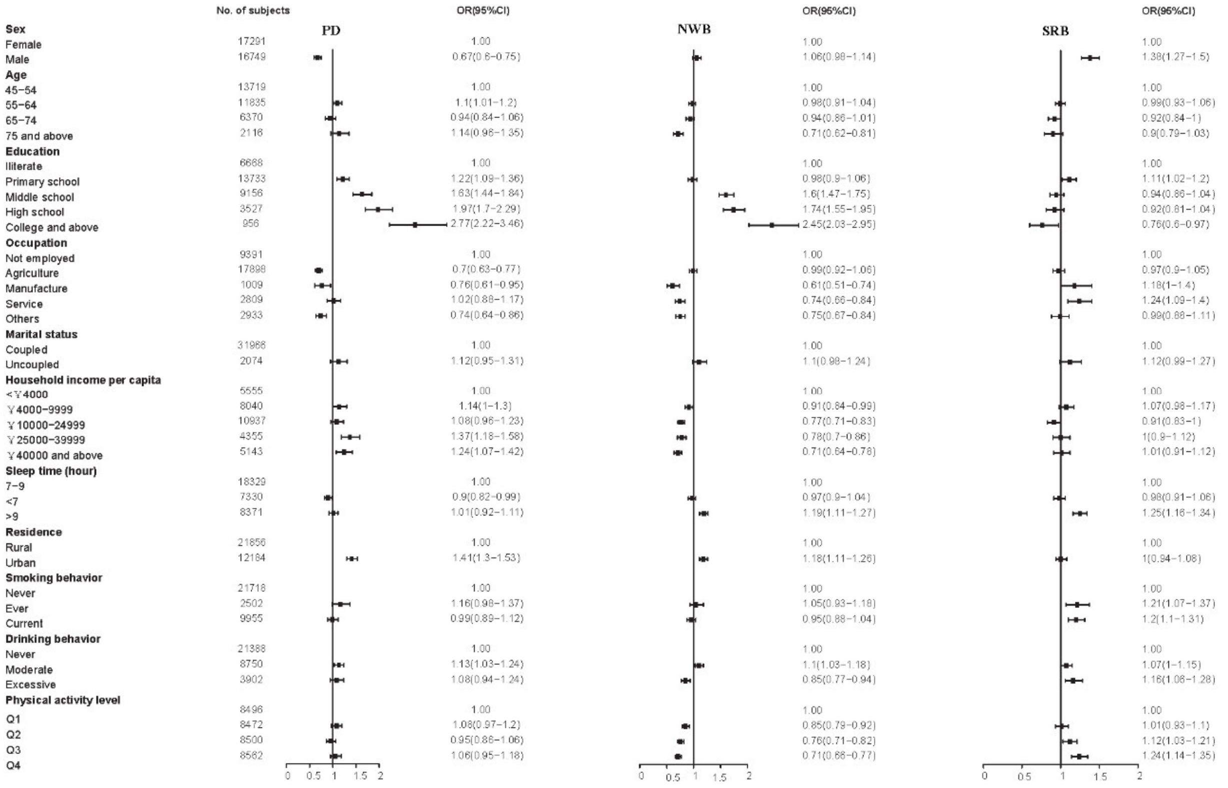

Figure S5. Estimated association of four dietary patterns with socioeconomic and lifestyle characteristics, among study population. CRB was the reference group. Abbreviation: CRB: common rice-based dietary pattern; PD: prudent diversified dietary pattern; NWB: Northern wheat-based dietary pattern; SRB: southern rice-based dietary pattern; OR: odds ratio; CI: confidence interval.

File S11. Association of physical activity with overweight/obesity and hypertension in study population and each dietary pattern.

Table S8 showed that higher physical activity level was associated lower risk of overweight/obesity and hypertension, and there were linear decreasing trends for the risk of overweight/obesity with the increase of physical activity level (P trend < 0.001). We further analyzed joint effect of dietary pattern and physical activity on the risk of overweight/obesity and hypertension, and found the associations of dietary pattern on overweight/obesity were modified by physical activity (P interaction <0.001). There were also linear decreasing trends along with the increase of physical activity level (P trend <0.001) in each dietary pattern. However, compared with SRB's subgroup of highest physical activity, NWB's subgroup of highest physical activity still had 31% higher risk of overweight/obesity. These indicates the relatively lower physical activity level for NWB subgroup (Table S7) might be another factor for the higher risk of overweight/obesity besides the suboptimal diet, while the independent effects of dietary pattern on overweight/obesity could not be ignored. Yet, higher physical activity appeared not to reduce the risk of hypertension linearly in study population, and the results did not support the modifying effects of physical activity on the association of dietary pattern on hypertension. Further investigation on this topic is warranted.

Table S9. Estimated associations between physical activity level and overweight/obesity and hypertension in study population and each dietary pattern *.

Outcomes	Modifier	Overall	NWB	CRB	PD	SRB
Overweight/obesity	Physical activity level					
	Q1	1.00	1.00	0.69 (0.62–0.77)	0.66 (0.56–0.77)	0.59(0.52–0.67)
	Q2	0.91 (0.86–0.97)	0.92 (0.82–1.03)	0.67(0.60–0.74)	0.55 (0.48–0.64)	0.56 (0.49–0.64)
	Q3	0.84 (0.79–0.90)	0.89 (0.79–1.01)	0.62 (0.56–0.69)	0.57 (0.48–0.67)	0.51 (0.45–0.58)
	Q4	0.71 (0.67–0.76)	0.78(0.67–0.91)	0.53 (0.46–0.61)	0.55 (0.45–0.68)	0.47(0.39–0.55)
	P_{trend} or $P_{interaction}$ [a]	<0.001	<0.001			
Hypertension	Physical activity level					
	Q1	1.00	1.00	0.91 (0.81–1.01)	0.84 (0.73–0.97)	0.81(0.71–0.93)
	Q2	0.92 (0.86–0.98)	0.93 (0.82–1.05)	0.88 (0.78–0.98)	0.88 (0.75–1.03)	0.78 (0.68–0.89)
	Q3	0.98 (0.92–1.04)	0.93 (0.80–1.09)	0.92 (0.82–1.03)	0.66 (0.57–0.77)	0.88 (0.77–1.00)
	Q4	0.92 (0.86–0.98)	0.91(0.81–1.01)	0.84 (0.73–0.97)	0.77 (0.64–0.93)	0.81 (0.69–0.94)
	P_{trend} or $P_{interaction}$ [a]	0.102	>0.129			

*. All models adjusted for were adjusted for sex, age, ethnic group, education level, household income per capita, occupation, smoking behavior, drinking behavior, physical activity, total energy intake, and family history of cardiovascular disease and diabetes, and body mass index were in addition adjusted for hypertension. [a.] The interaction of dietary patterns and quartiles of physical activity level were tested in above models.

File S12. Associations of protein food sources with overweight/obesity and hypertension in various dietary patterns.

Figure S4 indicates proportion of animal protein increases with the increase of percentage energy from protein in various dietary patterns. However, animal food sources contributing to the increased animal protein differs among different dietary patterns. The major animal protein foods for SRB are meat, fish, poultry, processed meat, and offal, but the increased part of animal protein are mainly contributed by fish, poultry, processed meat, and offal after percentage energy from protein exceeding 15%. The increased part of animal protein in CRB are mainly contributed by fish and poultry after percentage energy from protein exceeding 20%. In contrast, PD posits a decreasing trend of processed meat intake and increasing trend of dairy intake with the increase of percentage energy from protein, whereas there is a similar increasing trend of offal intake with SRB, which indicated PD had an increased proportion of dairy-sourced protein and a lower proportion of protein from processed meat than SRB with the increase of animal protein. In addition, meat, fish, and dairy contribute most to the increase of percentage energy from animal protein in NWB.

Table S8 shows that higher animal protein is not associated with overweight/obesity and negatively associated with hypertension (OR = 0.77,

95%CI: 0.72–0.83) in overall population, whereas plant protein is positively associated with overweight/obesity (OR = 1.15, 95%CI: 1.08–1.23) and not associated with hypertension (OR = 1.03, 95%CI: 0.97–1.12). However, the association differs in different dietary patterns. Higher animal protein intake is positively associated with overweight/obesity (OR = 1.17, 95%CI: 1.01–1.35) and not associated with hypertension (OR = 0.86, 95%CI: 0.75–1.01) in SRB's subgroup, but the positive association with overweight/obesity appeared to be not related to meat intake (OR = 0.73, 95%CI: 0.43–1.41), fish (OR = 1.10, 95%CI: 0.95–1.27), or poultry (OR = 0.96, 95%CI: 0.85–1.08), rather, it appears to be related to processed meat (OR = 1.27, 95%CI: 1.02–1.59) and offal intake (OR = 1.30, 95%CI: 1.06–1.58). In contrast, higher animal protein intake is not associated overweight/obesity in CRB (OR: 0.95 (0.86–1.05), PD (OR = 0.83, 95%CI: 0.68–1.02) and NWB (OR = 1.08, 95%CI: 0.95–1.23), but is negatively associated with hypertension in CRB (OR = 0.78, 95%CI: 0.70–0.87), PD (OR = 0.75, 95%CI: 0.60–0.94), and NWB (OR = 0.77, 95%CI: 0.67–0.88), and the negative association appears to be closely related to higher fish intake (OR = 0.79, 95%CI: 0.71–0.89) for CRB, higher poultry intake (OR = 0.78, 95%CI: 0.65–0.93) for PD, and higher dairy intake (OR = 0.61, 95%CI: 0.49–0.76) for NWB which might partly offset the increased risk for higher meat intake (OR = 1.18, 95%CI: 1.06–1.30). Of note, the results indicate no association of soybean intake with overweight/obesity and hypertension in overall participants and each subgroup.

Figure S4, Table S8, and Figure 6 suggest that the proportion of animal protein increases with the increase of percentage energy from protein, and dietary patterns with different animal protein foods composition will show different trends in the association between protein intake and overweight/obesity.

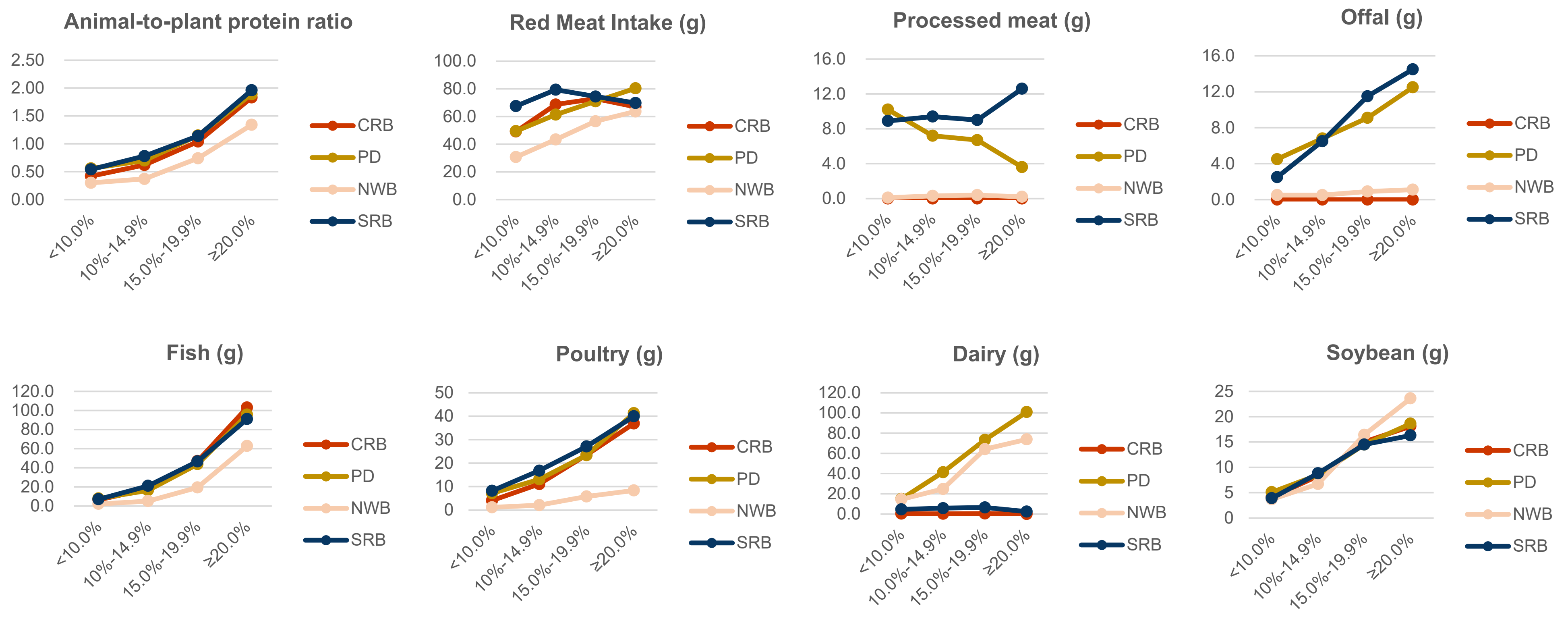

Figure S6. Analysis of protein food sources grouped by percentage energy from protein and dietary patterns. X axis represents four groups with increasing percentage energy from protein, and Y axis represents absolute intake (g) of each protein food group.

Table S10. Association of protein food sources with overweight/obesity and hypertension in overall population and various dietary patterns †.

	For overweight/obesity, OR(95%CI)					For hypertension, OR(95%CI)				
	Overall	CRB	PD	NWB	SRB	Overall	CRB	PD	NWB	SRB
Animal protein, %kcal										
Quartile4 vs. quartile1	1.01 (0.95–1.08)	0.95 (0.86–1.05)	0.83 (0.68–1.02)	1.08 (0.95–1.23)	1.17 (1.01–1.35) *	0.77 (0.72–0.83) *	0.78 (0.70–0.87) *	0.75 (0.60–0.94) *	0.77 (0.67–0.88) *	0.86 (0.75–1.01)
Plant protein, %kcal										
Quartile4 vs. quartile1	1.15 (1.08–1.23) *	1.15 (1.04–1.28) *	1.32 (1.09–1.60) *	1.08 (0.95–1.23)	1.14 (0.99–1.32)	1.03 (0.97–1.12)	0.99 (0.90–1.10)	1.21(0.98–1.48)	1.11(0.98–1.26)	0.95 (0.82–1.09)
Meat intake, g										
Group5 vs. group1 [a]	1.39 (0.87–2.23)	1.02 (0.94–1.11) [b]	0.82 (0.34–1.96)	0.95 (0.86–1.05) [b]	0.78 (0.43–1.41)	1.12 (0.69–1.82)	1.04 (0.95–1.13) [b]	1.11 (0.45–2.78)	1.18 (1.06–1.30) [b]*	0.77 (0.42–1.41)
Processed meat intake, g										
Group5 vs. group1 [a]	1.22 (1.01–1.48) *	--[c]	1.04 (0.70–1.52)	--	1.27 (1.02–1.59) *	1.23 (1.02–1.50) *	--	1.69 (1.12–2.53) *	--	1.12 (0.89–1.41)
Offal intake, g										
Group4 vs. group1 [a]	1.22 (1.04–1.42) *	--	1.04 (0.79–1.38)	--	1.30 (1.06–1.58) *	1.03 (0.87–1.20)	--	0.93 (0.70–1.25)	--	0.99 (0.81–1.22)
Dairy intake, g										
Group5 vs. group1 [a]	0.99 (0.85–1.16)	--	1.05 (0.82–1.37)	0.89 (0.72–1.10)	--	0.68 (0.58–0.80) *	--	0.87 (0.66–1.16)	0.61 (0.49–0.76) *	--
Poultry intake, g										
Group5 vs. group1 [a]	0.99 (0.93–1.06)	1.00 (0.91–1.09)	1.05 (0.89–1.23)	--	0.96 (0.85–1.08)	0.91 (0.86–0.97) *	0.96 (0.87–1.05)	0.78 (0.65–0.93) *	--	0.90(0.80–1.01)
Fish intake, g										
Group5 vs. group1 [a]	0.93 (0.86–1.00)	0.84 (0.76–0.94) *	0.83 (0.67–1.03)	1.08 (0.84–1.39)	1.10 (0.95–1.27)	0.90(0.83–0.97) *	0.79 (0.71–0.89) *	1.01 (0.81–1.27)	0.93 (0.72–1.20)	1.10 (0.95–1.28)

Soybean, g										
Group5 vs. group1 [a]	1.08 (1.00–1.16) [d]	1.06 (0.95–1.18)	0.98 (0.80–1.21)	1.16 (1.00–1.36) [d]	1.11 (0.95–1.29)	1.02 (0.95–1.10)	1.05 (0.94–1.17)	1.12(0.90–1.39)	0.94 (0.80–1.11)	1.02(0.88–1.20)

†. All models were adjusted for sex, age, ethnic group, education level, household income per capita, occupation, smoking behavior, drinking behavior, total energy intake, dark green vegetables, fresh fruits, physical activity, and family history of cardiovascular disease and diabetes.; models for hypertension were in addition adjusted for body mass index. *. $p < 0.05$; [a.] Grouped by actual intake; for meat, the cutoff values for group 1 to group 5 were 0, <25 g, 25–49 g, 50–74 g, 75–99 g, and >100 g, respectively; for processed meat, the cutoff values were 0, <15 g, 15–29 g, 30–59 g, and >60 g; for offal, they were 0, <20 g, 20–39 g, and >40 g; for dairy, they were 0, <50 g, 50–99 g, 100–199 g, and >200g; for poultry and fish, they were 0, <25 g, 25–49 g, 50–74 g, and >75 g; for soy, they were 0, <6 g, 6–11 g, 12–24 g, and >25 g. [b.] Only three groups in the corresponding pattern, so the contrast was group 3 vs group 1. [c.] People in the corresponding dietary pattern consumed too few relative protein foods, so the odds ratio was not estimated. [d.] No significance.
